# Supplementary material for: Arginine Auxotrophy Affects Siderophore Biosynthesis and Attenuates Virulence of Aspergillus fumigatus
Source: Genes (Basel). 2020 Apr 15;11(4):423. doi: 10.3390/genes11040423 (PMC7231135; doi:10.3390/genes11040423)
Supplement: Supplementary file 1 [file genes-11-00423-s001.pdf]

Supplementary Material

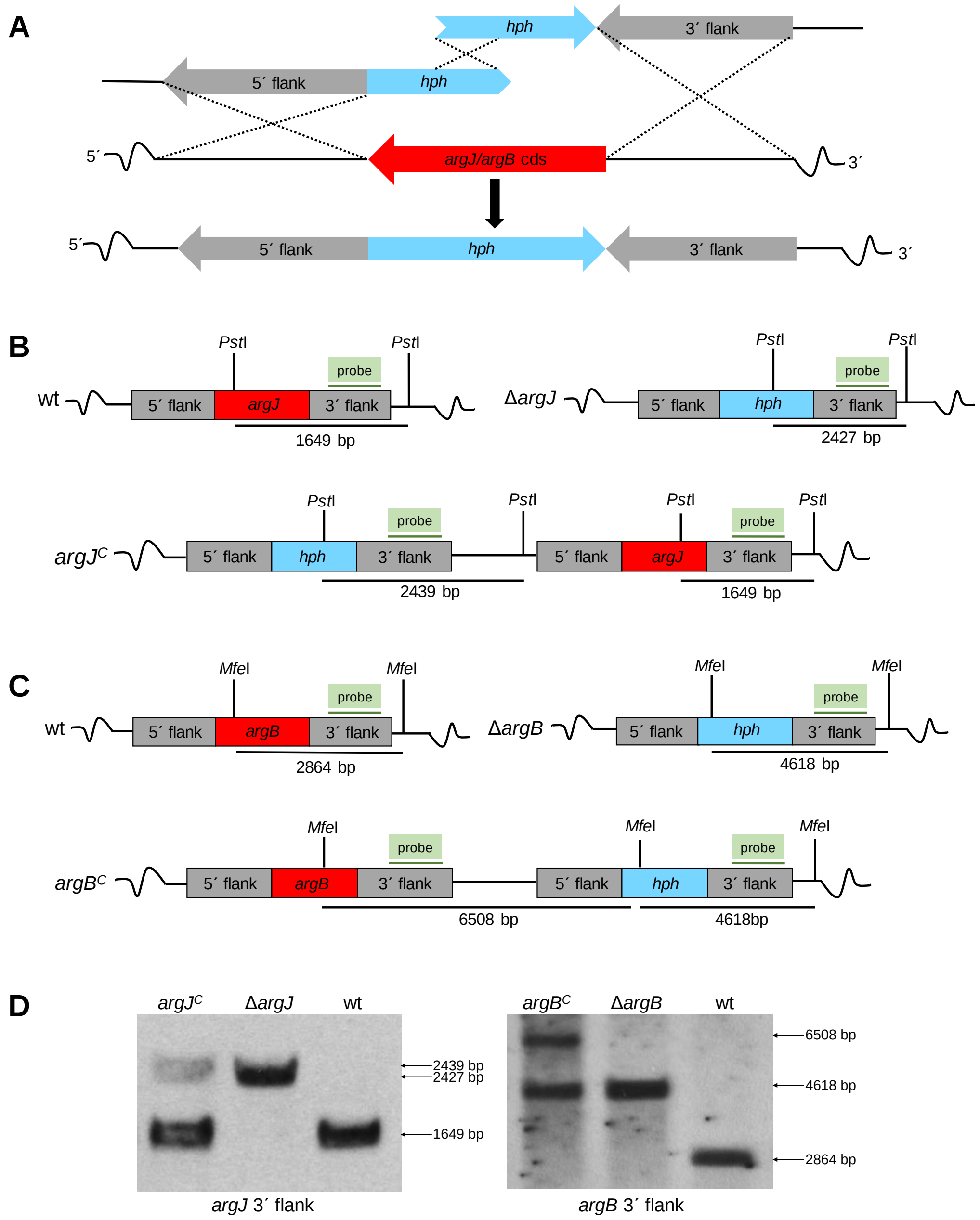

Supplementary Figure S1. Deletion and reconstitution of *argJ* and *argB* in *A. fumigatus*. (A) Schematic view of the split-marker technique-mediated deletion of *argJ* and *argB* in *A. fumigatus* wt (AfS77). (B) Genomic organization of the *argJ* locus in wt, Δ*argJ* and *argJ*^{C}. Genomic digestion with *Pst*I resulted in following fragments upon hybridization with a probe against the 3′ flank: 1.6 kb for the wt, 2.4 kb for Δ*argJ*, and 2.4 kb and 1.6 kb for *argJ*^{C}. (C) Genomic organization of the *argB* locus in wt, Δ*argB* and *argB*^{C}. Genomic digestion with *Mfe*I resulted in following fragments to be detected by a probe against the 3′ flank: 2.9 kb for the wt, 4.6 kb for *ΔargB*, and 6.5 kb and 4.6 kb for *argB*^{C}. (D) Southern blot analysis using the strategies outlined in (B) and (C) for *argJ* and *argB*, respectively.

Supplementary Table S1. Arginine biosynthetic genes in *A. fumigatus* and human homologs identified by BLASTP (blast.ncbi.nlm.nih.gov/Blast.cgi) searches.

**Enzyme name**	**Gene[1]**	***A. fumigatus***	***Homo sapiens***	**BLASTP E-value**	**Identity[3]**
acetylglutamate synthase	*arg2*	AFUA_2G11490	4K30_A	1e-13	34% (50/146)
acetylglutamate kinase and N-acetyl-gamma-glutamyl-phosphate reductase, bifunctional enzyme	*arg5,6/ argEF*	AFUA_6G02910	AAN76451.1	1e-28	25% (108/428)
acetylornithine aminotransferase	*arg8*	AFUA_2G12470	2CAN_A	6e-64	32% (126/396)
ornithine acetyltransferase	*arg7/ argJ*	AFUA_5G08120[2]	-	no similarity found	-
carbamoylphosphate synthase	*cpa1*	AFUA_5G06780	XP_005264612	5e-112	47% (177/380)
ornithine carbamoyltransferase	*arg3/ argB*	AFUA_4G07190[2]	BAA00161.1	1e-95	43% (151/350)
argininosuccinate synthase	*arg1*	AFUA_2G04310	NP_446464.1	4e-151	53% (218/408)
argininosuccinate lyase	*arg4*	AFUA_3G07790	NP_000039.2	3e-170	54% (236/440)

[1] *S. cerevisiae/Aspergillus* nomenclature
[2] deletion in *A. fumigatus* resulted in this study in arginine auxotrophy
[3] % (number of identical amino acids (aa) per total domain aa)

Supplementary Table S2. Primers used for generation of Δ*argJ*, Δ*argB*, *argJ*^{C} *and argB*^{C}. Nucleotides used for cloning/fusion PCR are shown in lower case.

**Primer**	**Sequence 5'-3'**
oAfargJ-5´f	agtgaattcgagctcggtacCACCGTCGCTATCCTCTATAG
oAfargJ-5´r	tgtacctaggGAATATCTTCAAAAACCGCCTTAAAC
oAfargJ-hph-f	gaagatattcCCTAGGTACAGAAGTCCAATTG
oAfargJ-hph-r	tttggctcaaTCTAGAAAGAAGGATTACCTC
oAfargJ-3´f	tctttctagaTTGAGCCAAAGGACATGTAC
oAfargJ-´3´r	ttacgccaagcttgcatgccGTCTGCTCTGTGCTCCAAC
oAfargB-5´f	agtgaattcgagctcggtacCGGTCTGCATCCAAGACAAATTTTC
oAfargB-5´r	attggacttcGGTGGATGAGGTTGAAATATGG
oAfargB-hph-f	ctcatccaccGAAGTCCAATTGCTTCCG
oAfargB-hph-r	cattcgatttTTTGGCTCAATCTAGAAAG
oAfargB-3´f	ttgagccaaaAAATCGAATGGTTTCTATAGAAAAAGCAAGCCGAAAC
oAfargB-3´r	ttacgccaagcttgcatgccGTCTCGGCGTCCGGCCGA
oAfagaA-f	5´-ACCGCACCAAAGAGCAAC
oAfagaA-r	5´-ACCGCACCAAAGAGCAAC
oAfargEF-f	5´-TTCGTACTCGCCATAGCC
oAfargEF-r	5´-CTTCTCAATGCTCACCCC
oAfamcA-f	5´-TCAATGGAGCTGCCTGTC
oAfamcA-r	5´-CAATTCCGTAGCCCTTCG
oAfsidA-f	5´-AACTACCTCCACCAGAAG
oAfsidA-r	5´-GAACGGCAATGTTGTAAG

Supplementary Table S3. Fungal strains used in this study.

**Strain**	**Description**	**Reference**
**AfS77 (wt)**	ATCC46645, *akuA*(AFUA_5G05680)*::loxP*	[33]
**Δ*argJ***	*argJ*(AFUA_5G08120)*::hph;* AfS77	This study
**Δ*argB***	*argB*(AFUA_4G07190)*::hph;* AfS77	This study
***argJ*^{C}**	*argJ::hph;* AfS77*; argJ*	This study
***argB*^{C}**	*argB::hph;* AfS77*; argB*	This study
